# Inhibition of TAZ impairs the migration ability of melanoma cells

**DOI:** 10.1515/biol-2022-0633

**Published:** 2023-06-21

**Authors:** Hao Zhang, Leijing Tu, Zhouji Ma, Yue Lin, Qian Tan

**Affiliations:** Department of Burns and Plastic Surgery, Nanjing Drum Tower Hospital, Affiliated Hospital of Medical School, Nanjing University, 321 Zhongshan Road, Nanjing 210008, China; Department of Burns and Plastic Surgery, Nanjing Drum Tower Hospital, Clinical College of Nanjing Medical University, Nanjing, China

**Keywords:** melanoma, metastasis, hippo signaling pathway, TAZ

## Abstract

Malignant melanoma (MM) is characterized by rapid growth, frequent metastasis, and high mortality. Targeted therapy for MM is still a research hotspot due to the increasing understanding of the hippo pathway. The aim of this study is to investigate the role of transcriptional coactivator with PDZ-binding motif (TAZ) in MM tumorigenesis. Based on the database analysis, we found that the median mRNA expression of TAZ (5.4) was found to be similar to that of YAP (5.5) in 473 human melanoma specimens. However, in 63 MM cell lines, the median expression of TAZ (10.8) was expressed at a higher level than that of YAP (9.5), which was then validated in A375. TAZ down-regulation by siRNA decreased the migration (72%) and invasion (74%) abilities of A375. Furthermore, the down-regulation of TAZ inhibited the proliferation of A375 without affecting apoptosis. We subsequently blocked hippo signaling with verteporfin and found that verteporfin application decreased the number of migrating (63%) and invading (69%) cells, respectively. We further found that Cyr61 declined following TAZ down-regulation. Moreover, TAZ negatively correlates with melanoma patient’s overall survival. Our data proved that TAZ contributed to MM metastasis, which might be a potential therapeutic target in the future.

## 1 Introduction

Malignant melanoma (MM) is a tumor originating from neural crest melanocytes and is characterized by rapid progression, high invasiveness, and is prone to distant metastasis [1]. Approximately 3% of MM patients lack an identifiable primary, which is also named melanoma of unknown primary (MUP). This rare MM remains biologically ill defined. Recent studies have shown that MUP patients appear to have a better prognosis than melanoma of known primary (MKP) patients, which is possibly due to higher immunogenicity as reflected in the immunologically mediated primary site regression [2]. Tumor metastasis is a significant contributing factor to the high mortality rate and the poor quality of life of advanced MM patients [3]. In China, the incidence of melanoma is increasing, with more than 10,000 new cases each year. Terminal MM patients have a poor prognosis with a median survival of only 6 months and a 5-year survival rate of less than 15%, which is due to the high metastasis rates of MM [4]. Thus, the lack of understanding of the mechanism underlying melanoma metastasis has hindered treatment for many years. With the development of molecular biology technology and MM-related molecular biology research, targeted therapies (BRAF inhibitors, KIT inhibitors, bevacizumab, and MEK inhibitors) have been slowly implemented in clinical trials, which have been approved by the US Food and Drug Administration (FDA) for the treatment of MM. However, there is still no drug targeting hippo signaling pathway.

The hippo signaling pathway is a series of evolutionarily conserved protein kinase cascades. It was first discovered in *Drosophila melanogaster* and has a similar signal transduction pathway in human cells [5]. The human homologous proteins are MST1/2, WW45, Lats, Mob1, Yes-associated protein (YAP), and its paralog, transcriptional coactivator with PDZ-binding motif (TAZ). In the hippo signaling pathway, MST1/2 binds to WW45 and phosphorylates Lats. Then, Lats binds to Mob1 and co-phosphorylates YAP/TAZ, which is inactivated after phosphorylation and inhibits the expression of downstream factors such as CTGF, Birc5, and Cyr61. Otherwise, YAP/TAZ translocates to the nucleus from the cytoplasm, where it binds the transcriptional co-activator TEADs, resulting in the corresponding biological effects [6]. The hippo signaling pathway plays an essential role in the regulation of cell growth, apoptosis, and tissue size. YAP/TAZ is the key downstream effector of the hippo signaling pathway. Abnormal expression of the core element of the hippo signaling pathway is closely related to the occurrence and development of tumors, which can cause a series of malignant biological behaviors such as proliferation, invasion, metastasis, and apoptosis escape of tumor cells [7,8]. The abnormal expression of YAP/TAZ exists in a variety of tumors, such as gastric cancer, prostate cancer, glioma, and melanoma [7,9–11]. Particularly, YAP has been proven to be an oncogene in MM. In 2020, Zhang et al. proved that YAP promoted the invasion of melanoma cells by targeting AXL, THBS1, and Cyr61 [12].

Few systematic research has been performed on TAZ, also called WW Domain Containing Transcription Regulator 1 (WWTR1) in MM, which is the paralog of YAP in the hippo signaling pathway. In recent years, TAZ has increasingly been recognized as an important transcription factor in human tumors. High TAZ expression has been found in a variety of human solid tumors, such as breast cancer, liver carcinomas, and glioma [9,13,14]. However, the role of TAZ in human MM has not been fully elucidated. Therefore, exploring the specific mechanism of TAZ in MM is of great significance for the molecular targeted therapy of MM. This article introduces TAZ and its role in MM tumorigenesis.

## 2 Materials and methods

### 2.1 Genomics analysis

Gene expression and overall survival (OS) analysis was performed on the R2 Microarray Analysis and Visualization platform (http://r2.amc.nl). Data on cutaneous melanoma and uveal melanoma were obtained from The Cancer Genome Atlas (TCGA). Melanoma cell lines were hybridized to Affymetrix Hu133_Plus 2 oligo arrays [15]. The cutoff values for the Kaplan–Meier analysis were determined using the online R2 microarray platform algorithm and were used to separate the high and low expression groups of genes. The expression cutoff for cutaneous melanoma was 434.5735 and that of uveal melanoma was 5.32. Informed consent has been obtained from all individuals included in this study according to the R2 Microarray Analysis and Visualization platform.

### 2.2 Cell culture

Human melanoma cell line A375 was provided by The Comprehensive Cancer Centre of Drum Tower Hospital. A375 cells were cultured in Dulbecco’s modified Eagle’s medium (Dulbecco’s modified eagle medium [DMEM], Life Technologies, Canada) supplemented with 10% fetal bovine serum (FBS, PAA Laboratories, Canada) and grown under 37°C, 5% CO_2_, and saturated humidity. When the culture reached the logarithmic growth phase and covered 90% of the flask bottom, the cells were passaged once. Subsequently, cells were transfected with non-targeting control or gene-specific siRNA using lipofectamine RNAiMAX (Invitrogen) according to the manufacturer’s instructions for small-interfering RNA (siRNA)-mediated downregulation. Three distinct, non-overlapping siRNA oligonucleotides targeting TAZ were as below (Control siRNA: AATTGTCCGAACGTGTCACGT; siTAZ: GGAUACAGGAGAAAACGCA; siTAZ2: AAACACCCAUGAACAUCAA; siTAZ3: AGGUACUUCCUCAAUCACA) [16,17].

### 2.3 Quantitative PCR (qPCR)

A375 cells were pretreated with siControl or siTAZ as described before. RNA was extracted from A375 melanoma cells using TRIzol reagent (Invitrogen, Carlsbad, CA) based on the protocol provided by the manufacturer. The cDNA library was generated by using a First-strand cDNA Kit (Roche Diagnostics). FastStart Universal SYBR Green Master was procured from Roche Diagnostics. After preparing the qPCR reaction solution, a real-time quantitative PCR machine (Applied Biosystems, USA) was used for amplification. The primers for human samples were: YAP-forward (F) 5′-cacagctcagcatcttcgac-3′, YAP-reverse (R) 5′-tattctgctgcactggtgga-3′; TAZ-forward (F) 5′-ggctgggagatgaccttcac-3′, TAZ-reverse (R) 5′-ctgagtggggtggttctgct-3′; GAPDH-forward (F): 5′-tgggtgtgaaccatgagaagtatg-3′, GAPDH-reverse (R), 5′-ggtgcaggaggcattgct-3′. The data were processed by the 2^−△△Ct^ method and normalized based on the expression of GAPDH.

### 2.4 Western blotting

A375 cells were pretreated with siControl or siTAZ as previously described. Protein lysates of A375 cells were quantified using the BCA Protein Assay Kit (Keygen) and an equal amount of protein samples (100 μg) was separated by SDS polyacrylamide gel electrophoresis SDS-PAGE (10%) and transferred to polyvinylidene difluoride membrane (Bio-Rad, USA) at 100 V constant pressure. The primary antibodies were added and incubated overnight at 4°C (<16 h). The following antibodies were used: mouse monoclonal anti-TAZ (Santa Cruz, TX, USA), rabbit monoclonal antiYAP (Abcam, Cambridge, MA, USA), mouse monoclonal anti-Cyr61 (Santa Cruz, TX, USA), mouse monoclonal anti-CTGF (Santa Cruz, TX, USA), mouse monoclonal anti-Birc5 (Santa Cruz, TX, USA), and anti-GAPDH (Cell Signaling, MA, USA). After incubation with horseradish peroxidase (HRP)-conjugated anti-rabbit or anti-mouse secondary antibodies (Abcam, Cambridge, MA, USA), the blot was developed with ECL substrate ECL reagent (Millipore, USA). The expression of the target protein was quantified by analyzing the density of the target band using Image-Pro Plus Software (Media Cybernetics, Inc, Rockville, MD, USA). All values were normalized to GAPDH.

### 2.5 Cell migration and invasion assays

A375 cells were pretreated with siControl or siTAZ as previously described. For Matrigel invasion assays, the Matrigel was dissolved on ice overnight and then diluted with DMEM medium at a ratio of 1:5. Then, the Matrigel was spread into the chamber at 100 μL/well and solidified at 37°C overnight. Cells in the logarithmic growth phase were collected and seeded in the chambers at a density of 5 × 10^4^ cells/200 μL per well. 500 μL DMEM containing 10% FBS was added to the lower chamber in a 24-well culture plate (Corning, USA). After the cells adhered to the Transwell insert, the upper chamber was replaced with serum-free DMEM. All groups were cultured in triplicate at 37°C, 5% CO_2_ in a humidified incubator. After 48 h incubation for the migration assay and 72 h incubation for the invasion assay, the chambers were removed and stained with 0.1% crystal violet for 15 min. The samples were washed with phosphate-buffered solution (PBS) and the upper chamber surface was gently wiped with a cotton swab. The invading cells were observed under a microscope (Leica, Germany) with a 20× objective in six random fields. Then, cell counting was performed to compare the migration and invasion index of the two groups of cells. The migration and invasion index was indicated to the number of cells divided by the mean of cells. Experiments were performed at least three times.

### 2.6 Cell proliferation analysis

A375 cells were pretreated with siControl or siTAZ as previously described. Cells in the logarithmic growth phase were collected and seeded into 96-well plates at a density of 5 × 10^3^ cells/well. After the adhesion of melanoma cells, the culture medium was replaced with serum-free DMEM. The experiments were carried out in triplicate. Subsequently, the plate was cultured in an incubator at 37°C and 5% CO_2_. 10 μL CCK8 working solution was added to each well before evaluation at 24, 48, and 72 h. After 2 h incubation, the absorbance value of each well was measured at a wavelength of 450 nm by the microplate reader. The standard curve was generated according to the manufacturer’s instructions (Beyotime Biotechnology).

### 2.7 Cell apoptosis

A375 cells were pretreated with siControl or siTAZ as described before. Cells in the logarithmic growth phase were collected and seeded into six-well plates at 15 × 10^5^ cells/well. After adhesion of the cells at 37°C, 5% CO_2_ in a humidified incubator, the culture medium was replaced by serum-free DMEM. The control group underwent the same treatment. After 6 h, the culture medium was replaced with DMEM containing 5% FBS. After 48 h, cells were harvested and gently washed twice with PBS. Annexin V/PI was added to each sample for staining according to the manufacturer’s instructions (BD company apoptosis kit) and then incubated at room temperature for 15 min before detection by using a BD FACS Aria™ II flow cytometer.

### 2.8 Immunofluorescence

A375 cells were pretreated with siControl or siTAZ as previously described. The cells were fixed with 4% paraformaldehyde diluted in PBS for 15 min at room temperature, perforated with 0.1% Triton X-100 diluted in PBS for 10 min at room temperature, and blocked with 1% bovine serum albumin for 60 min at room temperature. The primary antibodies mouse monoclonal anti-TAZ (Santa Cruz, TX, USA) were added to the slides and incubated at 4°C overnight. The slides were incubated at room temperature for 1 h with the corresponding secondary antibodies (Abcam, Cambridge, MA, USA), followed by DAPI staining. The images were taken under an immunofluorescence microscope (Leica, Germany). Signal intensity and colocalization were analyzed using ImageJ software.

### 2.9 Statistical analysis

The experimental results were statistically analyzed by SPSS16.0 (SPSS, Inc., Chicago, IL). Data are presented as mean ± standard error of the mean (SEM). The Student’s *t*-test was used for comparison between groups. The Kaplan–Meier method was used for survival analysis, and the Log-rank test was used to compare the survival time between the two groups. In this study, statistical significance was defined by *P < 0.05, **P < 0.01, ***P < 0.001.

## 3 Results

### 3.1 Expression of the hippo signaling pathway in melanoma

**Figure 1:**
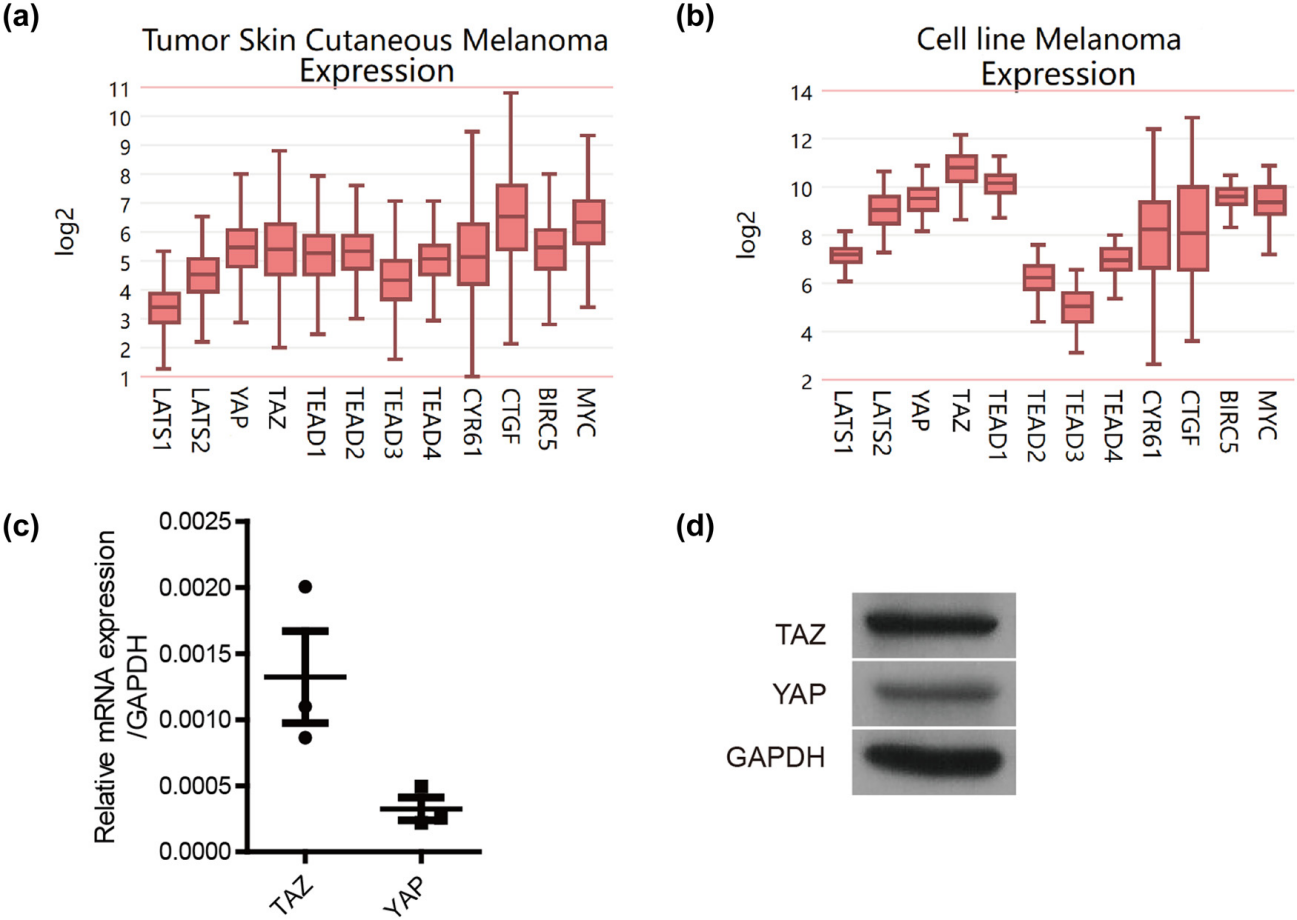
The expression of hippo signaling pathway in melanoma. (a) and (b) TCGA database shows the mRNA expression of hippo signaling pathway in human MM samples and MM cell lines. (c) and (d) The mRNA and protein expression level of TAZ was detected by qPCR and Western blot in A375 cell line.

To investigate the role of hippo signaling in MM, the mRNA expression of hippo core elements was first analyzed in 473 melanoma tissues and 63 melanoma cell lines on the R2 platform. In human melanoma tissue samples, the median expression of TAZ (5.4) was found to be similar to that of YAP (5.5) (Figure 1a). However, in MM cell lines, a higher median expression of TAZ (10.8) was observed compared to YAP (9.5) (Figure 1b). Subsequently, the A375 cell line was used for further validation. The mRNA and protein expression levels of TAZ were indeed higher than those of YAP in the A375 cell line (Figure 1c and d). Therefore, A375 cells were used to further investigate the role of TAZ in MM.

### 3.2 The role of TAZ in melanoma

**Figure 2:**
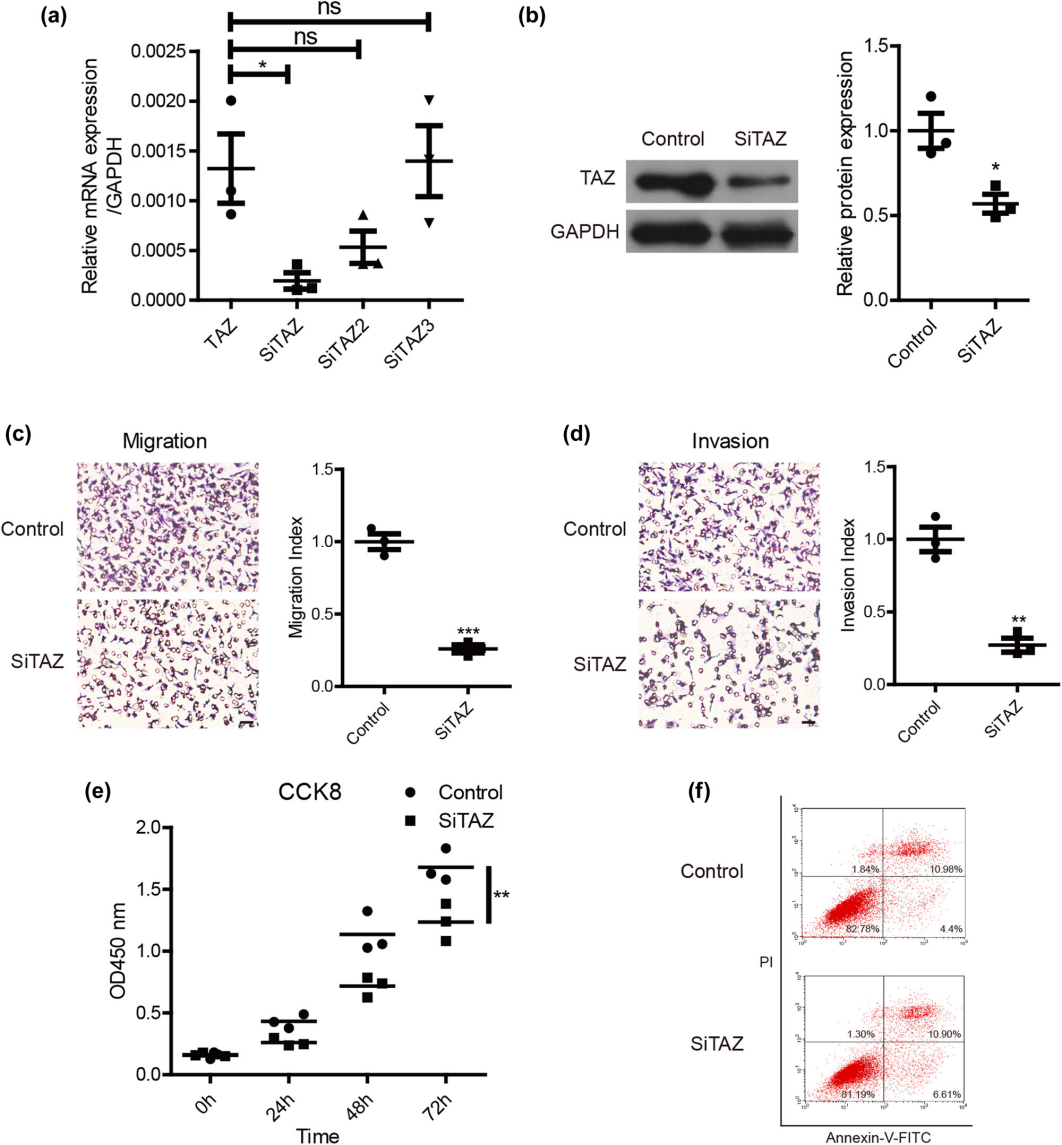
Down-regulation of TAZ inhibited the migration, invasion, and proliferation of melanoma cells. (a) The mRNA expression of TAZ in the group of control (Control siRNA), siTAZ, siTAZ2, and siTAZ3. (b) The protein expression of TAZ in the group of control and siTAZ. (c) After the down-regulation of TAZ, the number of migration cells was decreased compared with the control group (**P < 0.001). (d) After the down-regulation of TAZ, the number of invasion cells was decreased compared with the control group (**P < 0.01). (e) In the proliferation assay, cell proliferation was inhibited in the TAZ knockdown group compared with the control group at 72 h (**P < 0.01). (f) There was no significant difference in apoptosis between the siTAZ group and the control group.

siRNA was used to down-regulate the expression of TAZ to investigate the role of TAZ in melanoma. The qPCR results showed that siTAZ significantly suppressed the mRNA expression of TAZ (*P* < 0.05) in melanoma A375 cells (Figure 2a). Therefore, siTAZ was selected for subsequent experiments. Western blot analysis showed that SiTAZ treatment resulted in a significant decline in TAZ protein expression (43%, *P* < 0.05) (Figure 2b). Furthermore, the metastatic effect of TAZ was investigated by migration and invasion assays in A375 cells. The Transwell assay revealed a 70% decrease in the number of migration cells in the TAZ down-regulated group compared with the control group (*P* < 0.001) (Figure 2c). We also found a 74% decrease in the number of invasion cells in the TAZ down-regulation group compared with the control group (*P* < 0.01) (Figure 2d). Moreover, down-regulation of TAZ inhibited the proliferation of melanoma cells in the proliferation assay (*P* < 0.01) (Figure 2e). However, no significant change in cell apoptosis was observed after TAZ down-regulation (Figure 2f). These experiments all indicated that TAZ plays a vital role in melanoma tumorigenesis, especially metastasis.

**Figure 3:**
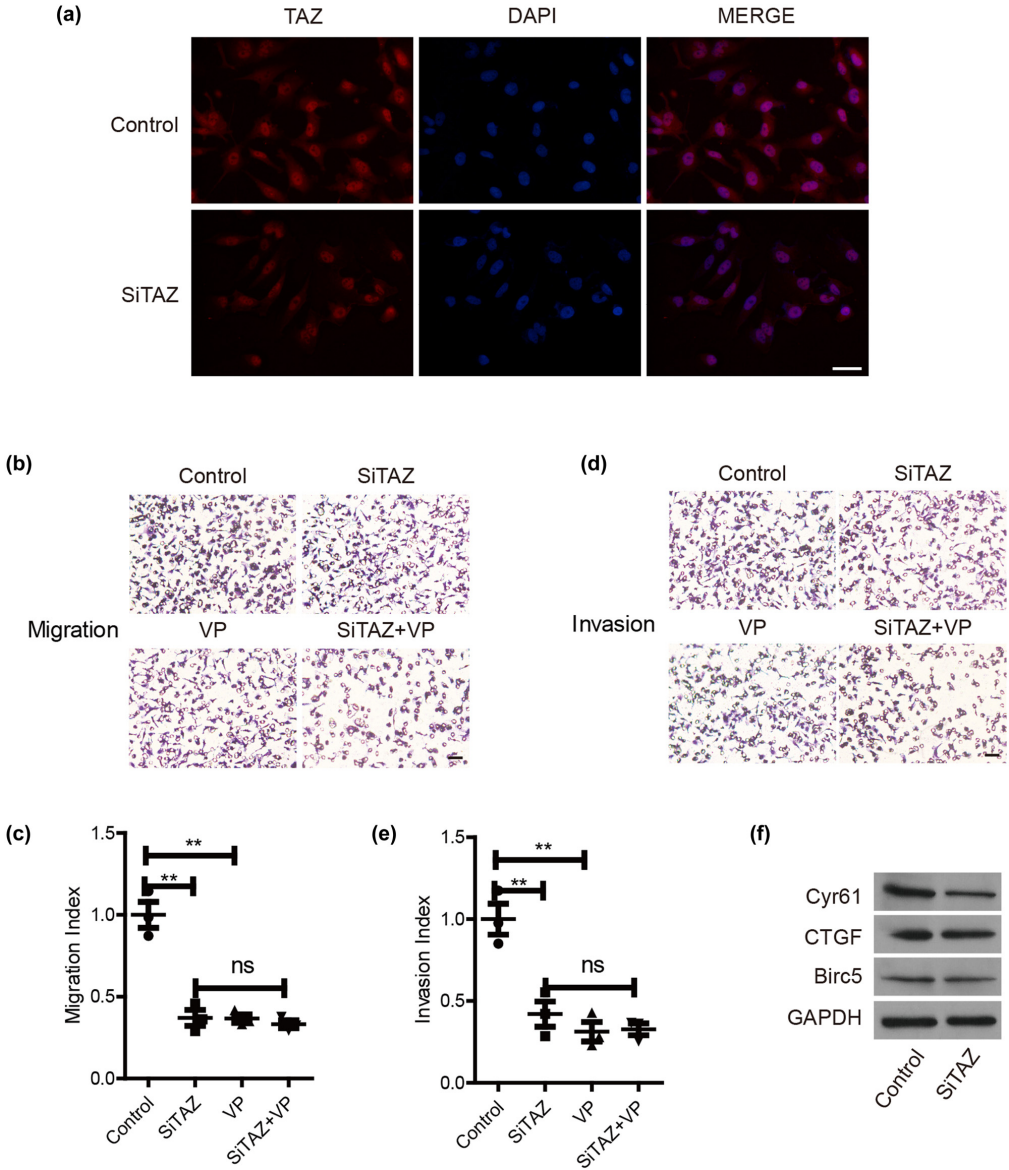
TAZ regulates melanoma metastasis through its downstream target gene. (a) IF was used to detect TAZ localization in A375 cells. (b and c) The number of migration cells was presented in the group of control, siTAZ, VP, and siTAZ + VP. (d and e) The number of invasion cells was presented in the group of control, siTAZ, VP, and siTAZ + VP. (f) The protein expression of Cyr61, CTGF, and Birc5 in the group of control and siTAZ.

To further investigate the mechanism of TAZ underlying melanoma cell metastasis, IF was first used to examine the localization of TAZ. We found that TAZ in cytoplasm and nucleus showed no big difference after TAZ down-regulation (Figure 3a). Verteporfin (VP), a YAP/TAZ inhibitor, effectively disrupted the YAP-TEAD interaction. Subsequently, the hippo signaling pathway was blocked with VP, and the results showed that VP treatment decreased the number of migrating A375 cells (63%, *P* < 0.01) (Figure 3b and c) and invading A375 cells (69%, *P* < 0.01) (Figure 3d and e). In the VP + siTAZ group, the number of migration and invasion cells decreased by 67% and 68%, respectively (Figure 3b–e). Compared with the siTAZ group, the VP + siTAZ group had no significant effect on cell metastasis (Figure 3b–e). In addition, the representative target genes of TAZ were examined after down-regulation in A375, revealing declined protein levels of Cyr61 (Figure 3f), which indicated that down-regulation of TAZ might inhibit tumor cell invasion and migration through Cyr61.

**Figure 4:**
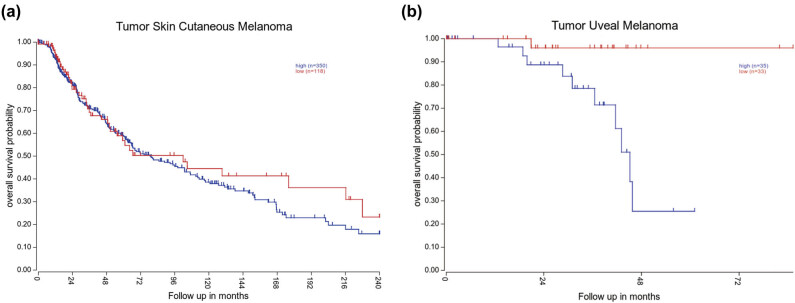
TAZ negatively correlates with melanoma patient’s OS. Association of TAZ expression with OS was presented by Kaplan–Meier plotter based on TCGA database of tumor skin cutaneous melanoma ((a) high, *n* = 350; low, *n* = 118; *P* = 0.396) and tumor uveal melanoma ((b) high, *n* = 35; low, *n* = 33; *P* < 0.05). The expression cutoff of tumor skin cutaneous melanoma was 434.5735 and that of tumor uveal melanoma was 5.32.

Both cutaneous melanoma and uveal melanoma originate from human melanocytes. Uveal melanoma is the most common primary intraocular malignant tumor in adults, accounting for 85% of all ocular melanomas. This disease is consistent with cutaneous melanoma in its high metastatic, refractory, and low survival rates. Therefore, we compared cutaneous melanoma and uveal melanoma. The R2 Microarray Analysis and Visualization Platform (http://r2.amc.nl) was used to further study the clinical significance of TAZ in melanoma patients. Based on the analysis of cutaneous melanoma, high TAZ expression was associated with lower OS (Figure 4a, TCGA: high, *n* = 350; low, *n* = 118; *P* = 0.396). Likewise, high TAZ expression was also associated with lower OS in uveal melanoma (Figure 4b, TCGA: high, *n* = 35; low, *n* = 33; *P* < 0.05), indicating that TAZ might have potential diagnostic, treatment, and prognosis applications in MM.

## 4 Discussion

In the past few years, the hippo signaling pathway has been widely explored in melanoma, but its precise mechanism remains to be elucidated [18]. This article found that the mutation or deletion of TAZ in the protein kinase cascade chain may affect the occurrence and development of melanoma.

The hippo pathway is an important signaling pathway that controls homeostasis. However, it has an inherent drawback: the mutation of a single gene can lead to dysregulation of the pathway and promote the occurrence of tumors. For organisms with a short life span, pathway disorders have no obvious consequences; but for organisms with a long life span, where cells are in constant renewal and complement, pathway disorders exert significant negative effects [6]. Therefore, it is important to clarify the regulation of the hippo pathway and TAZ in the development of MM.

During the growth process of tumors, after multiple divisions and proliferation, cells exhibit molecular biology or genetic changes, resulting in differences in tumor growth rate, invasion ability, drug sensitivity, prognosis, and other aspects. There are both oncogenic and non-oncogenic cell subpopulations in tumor tissue, reflecting the heterogeneity of tumor tissue. Normally, the human melanoma tissue is often a mixture, which includes melanoma cells, endothelial cells, fibroblasts, and so on, which can grow at the same time with the tumor cells. However, melanoma cell line is a type of cell. Thus, the expression levels of different genes differ between melanoma samples and cell lines. To explore whether TAZ plays a role in the tumorigenesis of melanoma, we used the TCGA database to predict its expression in human MM tissue samples (TCGA) and cell lines [15]. The A375 cell line was used to validate the expression of TAZ. Our results showed high TAZ expression in human MM tissue samples. In addition, the mRNA and protein expression of TAZ was higher than that of YAP in melanoma cell line A375. This finding is in accordance with Jason’s (2019) findings, which showed a higher TAZ protein expression than YAP in melanoma cell lines, such as A375, SKMEL5, and mel-537 [19]. The findings suggest that TAZ may play an important role in the occurrence and development of MM.

Moreover, the role of TAZ in melanoma tumorigenesis was explored. To study the effects of TAZ on melanoma metastasis, a Transwell assay was used to clarify the effect of TAZ on tumor cell invasion and migration. Our results showed a significant reduction in the invasion and migration ability of MM cells after TAZ down-regulation compared with the control group, indicating that TAZ can promote melanoma metastasis. Flore Nallet-Staub et al. also demonstrated that TAZ increases the invasiveness of melanoma by down-regulating TAZ in the 1205Lu melanoma cell line, which suppressed melanoma lung metastases in a mouse model [20]. In addition, Yap, the homolog of TAZ, has been confirmed to promote cell invasion in A375 [21], which is also in accordance with the present results. Furthermore, previous studies have reported that TAZ promotes cell invasion and migration in solid tumors and could be a reliable molecular target for gene therapy of MM. Surprisingly, TAZ was also found to boost melanoma cell proliferation without affecting cell apoptosis, which requires further research.

Our results showed that TAZ down-regulation could significantly inhibit the invasion and migration of MM cells. However, the mechanism underlying TAZ-mediated melanoma metastasis remains unclear. The Hippo signaling pathway comprises a series of protein kinase cascades, and a TAZ-dependent pathway in MM could provide a plausible explanation for this study’s findings. The results of this study suggest that down-regulation of TAZ can regulate the expression of Cyr61 and affect its downstream signaling, thereby affecting the invasion and migration of MM. Our results match those observed in earlier studies. For example, inhibition of Cyr61 prevented cell migration and invasion in melanoma A375 and B16 cell lines [22]. Long noncoding RNA CPS1-IT1 mediated melanoma cell migration and invasion by reducing Cyr61 expression [23]. Hence, a TAZ-Cyr61 axis could be hypothesized to participate in melanoma metastasis. Nevertheless, MM dissemination is an active process involving multiple steps, including the growth of transformed cells, cell dissociation of solid tumors, invasion of surrounding tissues, penetration of blood vessels, survival and accumulation in the circulation, penetration of the wall of bleeding vessels, entry into surrounding tissues, continued growth, and finally the formation of metastatic cancer [24]. Tumor invasion and metastasis can be divided into two aspects: ECM infiltration and vascular dissemination, and tumor cell homing and colonization [24,25]. Future studies should investigate how the TAZ-Cyr61 axis contributes to the invasion process.

Since YAP/TAZ is widely activated in MM, it is of great significance in the occurrence and development of tumors and shows great therapeutic potential [12]. However, due to technical limitations and the complex self-regulatory function of YAP/TAZ, it cannot be directly targeted to treat tumors [6]. The transcriptional co-regulators YAP/TAZ mainly pair with the TEAD transcription factor family to initiate gene expression signatures that are important in cancer development, progression, and metastasis [26]. Thus, YAP/TAZ-TEAD inhibition represents a promising novel cancer treatment option. It is exciting that many drugs that have entered the clinic restrict and several novel YAP/TAZ inhibitors are currently being developed [8,26]. In this study, the application of verteporfin (VP) impaired melanoma cell migration and invasion, supporting the role of the TAZ-Cyr61 axis in melanoma metastasis.

Gene therapy for MM is a current research hotspot due to the poor response of MM to traditional surgical resection, chemotherapy, and radiotherapy [27,28]. Novel therapies that have been approved by the FDA and the EMA are including immune checkpoint inhibitors, oncolytic immunotherapies, and targeted therapies (BRAF and MEK inhibitors). DNA markers such as BRAF and NRAS can predict response to target therapy and provide better options for patients. ctDNA and miRNA or lncRNA provide vital insights into the genetics of tumors, contribute to the understanding of disease pathophysiology, and have the great advantage of continuous, noninvasive sampling for disease monitoring [29]. The increase or loss of TAZ expression in MM may interfere with the metastasis of MM by regulating the hippo signaling pathway. At present, the treatment of advanced MM still mainly relies on immunotherapy and targeted therapy. In future studies, hippo intervention will be considered to verify the role of hippo in MM metastasis and to evaluate the potential of YAP/TAZ as a therapeutic target for advanced MM patients. Although the research on the hippo signaling pathway is still in its preliminary stage, YAP/TAZ is expected to become a marker for the early prediction of MM metastasis, which is helpful for the early diagnosis and treatment of MM.
